# Iron Content of Commercially Available Infant and Toddler Foods in the United States, 2015 †

**DOI:** 10.3390/nu12082439

**Published:** 2020-08-13

**Authors:** Marlana Bates, Priya M. Gupta, Mary E. Cogswell, Heather C. Hamner, Cria G. Perrine

**Affiliations:** 1 Division for Heart Disease and Stroke Prevention, National Center for Chronic Disease Prevention and Health Promotion, Centers for Disease Control and Prevention, Atlanta, GA 30341-3717, USA; mec0@cdc.gov; 2 Oak Ridge Institute for Science and Education (ORISE), Oak Ridge, TN 37831-0117, USA; 3 Hubert Department of Global Health, Rollins School of Public Health Emory University, Atlanta, GA 30322-4201, USA; pmgupta@emory.edu; 4 Division of Nutrition, Physical Activity, and Obesity, National Center for Chronic Disease Prevention and Health Promotion, Centers for Disease Control and Prevention, Atlanta, GA 30341-3717, USA; hfc2@cdc.gov (H.C.H.); cperrine@cdc.gov (C.G.P.)

**Keywords:** iron, infant, toddler, nutrition, complementary foods

## Abstract

Objectives: To describe the iron content of commercially available infant and toddler foods. Methods: Nutrition Facts label data were used from a 2015 database of 1037 commercial infant and toddler food and drink products. Products were grouped into food categories on the basis of name, ingredients, target age, and reference amounts customarily consumed (RACC). Mean and median iron content per 100 g and per RACC were calculated. The proportion of products considered good and excellent sources of iron were determined on the basis of percent daily value (% DV) thresholds. Results: Among products marketed for infants (aged 4–12 months), infant cereals had the highest mean (6.19 mg iron per RACC; 41.25 iron mg per 100 g) iron content. Among products marketed for toddlers (aged 12–36 months), vegetable-based mixtures or meals contained the highest mean iron in mg per RACC (mean: 2.97 mg) and dry, grain-based desserts had the highest mean iron in mg per 100 g (mean: 6.45 mg). Juice and drink products had the lowest mean iron contents in both infant and toddler products. Conclusions: Most commercially available infant cereals are considered to be an excellent source of iron, likely from fortification, but wide variability was observed in iron content by food category. Products that are considered good or excellent sources of iron (≥10% DV) can help consumers identify products with higher iron content, such as infant cereals or toddler vegetable-based mixtures/meals.

## 1. Introduction

Iron is an essential micronutrient across all stages of human development, but especially during a child’s first 1000 days (i.e., from conception through the first two years of life) [1,2]. Iron is required for normal infant growth and for cognitive development [3]. Iron deficiency among infants and toddlers can result in anemia and increased rates of mortality [4,5]. Additionally, the presence of inadequate iron stores is common in preterm infants [6,7,8]. Iron supplementation in exclusively breastfed infants has been recommended, since breastmilk may not provide sufficient iron content [8,9]. However, this generalized recommendation for exclusively breastfed infants is a matter of concern for some experts [10,11,12]. The American Academy of Pediatrics (AAP) recommends the introduction of complementary foods at around six months of age and encourages the consumption of those foods that provide key nutrients, such as iron [13]. From infancy into early childhood, recommended daily iron needs change, reflected by the iron dietary reference intake (DRI) values by life-stage group [14]. Infants aged 7–12 months have an estimated average requirement (EAR) of 6.9 mg of iron per day and a recommended dietary allowance (RDA) of 11 mg of iron per day [14]. However, toddlers and preschool age children aged one to three years have an EAR value of 3.0 mg of iron per day and an RDA value of 7.0 mg of iron per day [14]. Ensuring adequate iron nutrition during early childhood can help meet the needs of a growing child and reduce the risk of micronutrient deficiencies and impaired growth [8,13].

Previous studies examined iron status among toddlers [15], dietary intake of US infants and toddlers, including iron [16,17], and the leading sources of iron for infants and toddlers, including commercial infant toddler foods [18]. A few studies also examined the consumption of commercially prepared infant and toddler foods in the US [19,20]. Just over half of infants between 6 and 8 months of age and over a third of infants 9 to 11 months of age consumed commercially prepared baby-food fruits and vegetables in the 2008 Feeding Infants and Toddlers Study (FITS) [19]. Commercially available baby food dinners were consumed by 25% of 6 to 8 month-old and 9 to 11 month-old infants, as well as by 10% of 12 to 14 month-old toddlers [19]. Similar findings were observed utilizing National Health and Nutrition Examination Survey (NHANES) data from 2009 to 2014 in 6 to 11 month-olds [20]. A number of studies reported the iron content of current, commercially available infant or toddler food and drink products available internationally [21,22,23], but we found no study that reported the current iron contents of commercial US infant or toddler foods. With the known prevalent usage of commercially available infant and toddler foods in the US, awareness of the current iron contents of these products may help caretakers and health providers make more informed choices and recommendations about which foods provide higher levels of iron to ensure adequate iron intake among infants and toddlers. In addition, this knowledge can show food manufacturers those food categories with room for improvement regarding iron content. This study describes the iron contents of packaged infant and toddler food and drink products sold in the United States in 2015.

## 2. Materials and Methods

Nutrition Facts (NF) label data, including the nutrient contents and ingredient lists of complementary infant and toddler food and drink products sold in the United States, were collected from major grocery vendors and manufacturer websites and entered into a database during May–July 2015. The details of the data collection are reported elsewhere [24]. Briefly, 24 brands, accounting for >95% of the market share of infant and toddler food sales in the United States and Canada, were identified for inclusion on the basis of their availability in 11 stores in Atlanta, Georgia, and Seattle, Washington [24]. From these brands, products were identified from manufacturer websites, and NF label data were collected. If label data were not available online, then information was collected from products on-site at stores [24]. Products were included that contained the words “baby,” “infant,” “toddler,” or “tot,” or that had a clearly labeled age or developmental stage in the 0–36-month range. Products in developmental stages 1–3 were aimed at infants (aged 4–12 months), and stage 4 products were aimed at toddlers (aged 12–36 months) [24]. The final database included data on the nutrition content of 1037 commercial infant and toddler food and drink products.

Of the 1037 commercial infant and toddler food and drink products in the database, 1016 (98%) food products reported a percent of the daily value (% DV) for iron. The values are based on the age-specific daily recommendations for intake of key nutrients, i.e., 15 mg of iron for infants and 10 mg of iron for toddlers [25]. In addition, these values are used for consumers as a reference to determine the percentage a serving of food contributes to their daily intake of specific nutrients [25,26]. Products with <2% DV for iron (i.e., <0.3 mg per serving for infants or <0.2 mg for toddlers) are not required to list the amount of iron on the NF label, but they are required to include a statement indicating the food is not a significant source of iron [27].

Twenty-one applesauce squeeze-pouch products were missing a % DV for iron. There were five similar applesauce products with iron data in the database; of these, two contained 1% of the DV for iron, and three contained 2% of the DV for iron. A value of 2% DV for the 21 missing products was imputed, and the mean iron content was shown to be similar whether these 21 products were included or excluded from the analysis. Therefore, the 21 imputed values were included in the analysis, yielding 1037 products with available data on iron content.

Products were grouped into food categories on the basis of product name, ingredient list, target age (infants or toddlers), and the food categories used to determine the US Food and Drug Administration’s (FDA) reference amounts customarily consumed per eating occasion (RACC) (Table S1) [24]. RACCs are defined as the “serving sizes of foods that can be reasonably consumed at one eating occasion” [28]. RACCs are used to guide, but are not necessarily equal to the serving size listed by the manufacturer on the NF label [28].

Earlier studies using the 2012 and 2015 databases reported 13 food categories [24,29]. For this study, a few of the 13 food categories were combined or recategorized to increase sample size because of presumed similarity in iron concentration (mg per 100 g). For example, the food category “fruits only” combined fruit products targeting both infants and toddlers, which was previously separately reported [24]. Similar modifications were also made for pasta-based, vegetable-based, and meat-based meals or snacks categories. The toddler “sides” food category was excluded from the original 2015 analysis but included in the current analysis, though recategorized, as shown in Table S2 [24]. These side dish products were included in the vegetables only (*n* = 2) and meat-based meals or snacks (*n* = 3) categories. The food category modifications resulted in 13 separate food categories without distinguishing between infant or toddler targeted foods, as performed in previous papers (Table S2) [24,29]. Products missing data on stage (*n* = 15) or RACC values (*n* = 5) were excluded, resulting in a final analytical sample of 1017 products (*n* = 725 infant products and *n* = 292 toddler products).

Iron was expressed per RACC to standardize the serving size, and per 100 g. Because the amount of iron in milligrams is not presented on the NF label, this value was calculated by using the labeled % DVs. For these calculations, % DVs were first converted to milligrams, (e.g., 20% DV for iron, as reported on the NF label for a food targeted to infants, equals 0.20 × 15 mg, or 3.0 mg per serving). The amount per RACC or per 100 g was then determined on the basis of the serving size (e.g., if the serving size was listed as 50 g, and the RACC was 75 mg, then a product with 3.0 mg iron per serving would contain 4.5 mg iron per RACC and 6 mg iron per 100 g). Infant and toddler products were categorized as “good” or “excellent” sources of iron by using the FDA standards for nutrient content claims. The FDA defines a “good” source of iron as one with 10–19% of the DV for iron and an “excellent” source as one with 20% or more of the DV for iron [30]. The iron contents of infant cereals were also categorized into those that met the Special Supplemental Nutrition Program for Women, Infants, and Children (WIC) regulatory requirements of 45 mg of iron per 100 g of dry cereal, and those that did not [31].

The percentage of products considered to be “good” and “excellent” sources of iron was calculated overall and for each of the 13 food groups. The mean, standard error, median, and interquartile range of iron content were calculated for each food category. Presenting both the mean and median allowed for the description of the distribution of the iron content within food groups. Analyses were stratified by target age, as listed by the manufacturer, for foods marketed to infants (aged 4–12 months) and toddlers (aged 12–36 months). All statistical analyses were performed using SAS version 9.4 (SAS Institute, Inc., Cary, NC, USA).

## 3. Results

In general, most (>80%) infant and toddler products were not considered good or excellent sources of iron (i.e., containing <10% of the DV for iron). Of the overall sample of products, 7.6% were considered a good source and 8.3% an excellent source of iron, according to their reported % DVs (Table 1). Just over half of dry, grain-based desserts and about 46% of savory snacks were a good source of iron. Within only infant products, these proportions increased, with about 63% of dry grain-based desserts and 69% of savory snacks considered a good source of iron. Within toddler products, almost half of cereal bars and breakfast pastries were excellent sources of iron, and about a third of products in the fruits and grains, savory snacks, and dry, fruit-based desserts categories were good sources of iron. Excellent sources of iron were most common in the infant cereal, dry and instant (82.5%), and toddler vegetable-based mixtures or meals (75.0%) categories.

### 3.1. Infants

#### 3.1.1. Iron Content Per RACC

Among the 725 products marketed for infants, infant cereals had the highest mean (6.19 mg ± 0.49) iron content of all food categories examined in mg per RACC. Savory snacks and dry, grain-based desserts had the second and third highest mean iron contents in mg per RACC among the food product categories (3.21 mg ± 0.63 and 1.38 mg ± 0.12, respectively) (Table 2). Although the mean iron content of fruit and grain products was 1.32 mg per RACC, the median was 0.33 mg per RACC, indicating a skewed distribution with variability of iron content across products. Of categories with products containing more than 0 mg of iron, fruit products had the lowest mean iron contents in mg per RACC (0.31 mg ± 0.02). Infant dry, fruit-based snacks and juice drinks did not contain any iron.

#### 3.1.2. Iron Content Per 100 g

Comparable to the results of evaluating iron content per RACC, infant cereals (mean: 41.25 mg ± 3.29), savory snacks (mean: 21.43 mg ± 4.20), and dry, grain-based desserts (mean: 19.74 mg ± 1.69) had the highest iron contents in mg per 100 g of the 13 food categories (Table 2). Fruit-only products (0.29 mg ± 0.02) had the lowest mean iron content in mg per 100 g. About three in four (*n* = 29) infant cereals met the WIC requirement of 45 mg of iron per 100 g of dry cereal (data not shown). The 11 cereals that did not meet the WIC iron requirements contained iron ranging from 0–43 mg per 100 g of dry cereal (data not shown).

### 3.2. Toddlers

#### 3.2.1. Iron Content Per RACC

Among the 292 products marketed for toddlers, the three food categories containing the highest mean iron in mg per RACC were vegetable-based mixtures (2.97 mg ± 0.71), pasta-based meals or snacks (1.92 mg ± 0.31), and cereal bars and breakfast pastries (1.22 mg ± 0.15) (Table 3). For those categories with skewed distributions of iron content, including savory snacks and dry, fruit-based snacks, the median iron contents per RACC were 0.36 mg (IQR: 1.10) and 0.21 mg (IQR: 0.38), respectively. Juice or drink products had the lowest mean (0.10 mg ± 0.02) iron content in mg per RACC.

#### 3.2.2. Iron Content Per 100 g

The three categories with the highest mean iron content in mg per 100 g servings were shown to be dry, grain-based desserts (6.45 mg ± 1.08), cereal bars and breakfast pastries (6.10 mg ± 0.74), and savory snacks (5.18 mg ± 1.28) (Table 3). Similar to the reported iron content per RACC, savory snacks and dry, fruit-based snacks showed skewed distributions within the food categories in mg of iron per 100 g serving, with medians of 2.38 mg (IQR: 7.33) and 1.43 mg (IQR: 2.54), respectively. Juice and drink products had the lowest mean (0.08 mg ± 0.02) iron content in mg per 100 g.

## 4. Discussion

This study provides comprehensive data on the iron contents of commercially available infant and toddler food products sold in the United States in 2015. Of products targeted at infants, infant cereals, dry, grain-based desserts, and savory snacks contained the highest iron contents in mg per RACC and per 100 g. Of products targeted at toddlers, vegetable-based mixtures or meals, pasta-based meals or snacks, and cereal bars and breakfast pastries had the highest iron contents in mg per RACC. Although toddler vegetable-based mixtures or meals and pasta-based meals or snacks had the highest iron content per RACC, they were noticeably lower per 100 g because of an RACC value of 170 g for each. The RACC is much smaller in other food categories, including toddler cereal bars and breakfast pastries (20 g) and dry, grain-based desserts (15 g), leading to very high iron content per 100 g. These toddler product categories with the highest iron concentrations (mg per 100 g) may include fortificants, such as ferrous sulfate, in the ingredient list (e.g., dry, grain-based desserts); however, these products are typically consumed in servings less than 100 g, and can contain other ingredients, such as sugar, salt, or fat, which should be limited in the diet [9,24].

Unexpectedly, commercially available meat-based meals or mixtures did not contain high amounts of iron relative to other food categories. Krebs and colleagues found that commercially available mixed dinners combining a vegetable or starch with a meat source contain lower amounts of iron than single-ingredient, pureed meats [32]. Past data from the Feeding Infants and Toddlers study showed that chicken and turkey products are higher in iron than meat products with added ingredients (i.e., hot dogs, sausages, and cold cuts) [33]. Both the AAP and WIC recommend the introduction of single-ingredient, complementary foods [34,35]. Although this recommendation is often discussed within the context of food allergies, food tolerance, and food acceptance, this strategy may also be advantageous to explore, specifically for meat-based products, to ensure that young children receive adequate intake of micronutrients such as iron. In addition, because the percentage of ingredients in each product was unknown, there was no way to assess the bioavailability of the iron contents of infant and toddler food and drink products from the database used for this paper (i.e., heme and nonheme iron; plant vs. animal sources of iron) [36].

A 2003 study by Lutter and Dewey proposed the recommended nutrient content of fortified complementary foods per 100 g [37]. The investigators recommended that iron be present at 14 mg per 100 g in fortified complementary food products marketed for children aged 6–23 months [37]. The results of this study suggest that, on average, infant products, including cereals, savory snacks, and dry, grain-based desserts, meet this standard (14 mg per 100 g); however, some products, such as snacks and desserts, may contain ingredients that should be limited [24]. The other infant product categories and products marketed for toddlers currently fall short of this recommendation. However, the results presented in this study only show the mean and median values by food category; individual products may be above or below this threshold.

With the knowledge that the market share of most infant and toddler foods in 2015 is included in the utilized database by brand, this study contains comprehensive data on the iron contents of commercially available infant and toddler foods available in the United States. The study is subject to limitations. First, iron % DVs are based on the Nutrition Facts label. The FDA regulatory standards state that the nutrient content of a product can vary from what is reported on the label because of rounding and allowance for possible error in laboratory analyses of food products [27]. Next, this analysis was limited to describing the nutrient contents of infant and toddler products and did not include information on consumption or intake. Lastly, data on food products were collected from grocery stores and manufacturer websites for brands of infant and toddler products in 2015. It is expected that since the data were collected, some infant and toddler products on the market have been added, reformulated, or removed. Although this database is comprehensive, it may not be representative of the products currently on the market. For this reason, the generalizability of our results should be approached with caution. Future research may include a more frequent scan of the market to look at trends in food and drink product data.

## 5. Conclusions

Among packaged foods produced for infants and toddlers in 2015, there was wide variability in mean iron content among food categories, both per RACC and per 100 g. Although some food categories had a high proportion of products with 10% or more of the DV per serving, most products marketed to infants and toddlers were not considered a good or excellent source of iron. Despite these findings, if parents and caretakers utilizing commercially available foods choose iron-rich products, such as many infant cereals or toddler vegetable-based mixtures or meals products, then adequate iron of iron could be achieved in infants and toddlers on the basis of the current micronutrient DRIs. Understanding the iron contents of packaged foods and the dietary intake of infants and toddlers (including from non-commercially prepared foods) would help reveal gaps in the nutrient compositions of packaged foods.

## Figures and Tables

**Table 1:** Proportion of infant ^a^ and toddler ^a^ food or drink products considered good ^b^ and excellent ^c^ sources of iron on the basis of daily value (DV) ^d^ by food category, 2015.

Food Product Category	All Products			Infant Products			Toddler Products		
	*N*	Good Source	Excellent Source	*N*	Good Source	Excellent Source	*N*	Good Source	Excellent Source
		*n* (%)	*n* (%)		*n* (%)	*n* (%)		*n* (%)	*n* (%)
All products	1017	77 (7.6)	84 (8.3)	725	44 (6.1)	48 (6.6)	292	33 (11.3)	36 (12.3)
Cereals, dry and instant	40	-	33 (82.5)	40	-	33 (82.5)	-	-	-
Vegetables only	52	-	-	52	-	-	-	-	-
Fruits only	305	3 (1.0)	2 (0.7)	256	-	1 (0.4)	49	3 (6.1)	1 (2.0)
Fruits and grains	78	4 (5.1)	13 (16.7)	66	-	12 (18.2)	12	4 (33.3)	1 (8.3)
Dairy-based	100	-	1 (1.0)	41	-	1 (2.4)	59	-	-
Meat-based meals or snacks	96	5 (5.2)	1 (1.0)	74	2 (2.7)	-	22	3 (13.6)	1 (4.5)
Pasta-based meals or snacks	28	3 (10.7)	7 (25.0)	11	3 (27.3)	-	17	-	7 (41.2)
Vegetable-based mixtures or meals	108	4 (3.7)	4 (3.7)	104	3 (2.9)	1 (1.0)	4	1 (25.0)	3 (75.0)
Cereal bars and breakfast pastries	29	2 (6.9)	14 (48.3)	-	-	-	29	2 (6.9)	14 (48.3)
Savory snacks	33	15 (45.5)	3 (9.1)	13	9 (69.2)	-	20	6 (30.0)	3 (15.0)
Dry, grain-based desserts	76	39 (51.3)	5 (6.6)	43	27 (62.8)	-	33	12 (36.4)	5 (15.2)
Dry, fruit-based snacks	47	2 (4.3)	1 (2.1)	11	-	-	36	2 (5.6)	1 (2.8)
Juice or drinks	25	-	-	14	-	-	11	-	-

^a^ Infant and toddler food and drink products included those marketed for children aged 4–12 months and 12–36 months, respectively. ^b^ The Food and Drug Administration (FDA) defines a good source of iron as one with 10–19% of the DV for iron. ^c^ The FDA defines an excellent source of iron as one with ≥20% of the DV for iron. ^d^ Daily values (DV) are units developed by the US Food and Drug Administration to help consumers determine the levels of nutrients in a standard serving of food in relation to the estimated need. No food products met the criteria.

**Table 2:** Iron content per reference amount customarily consumed (RACC) and per 100 g of infant ^a^ food and drink products by food product category, 2015.

Food Product Category	Iron (mg)/RACC ^b^				
	*N* = 725	Mean	SE ^c^	Median	IQR ^d^
Cereals, dry and instant	40	6.19	0.49	6.75	1.18
Vegetables only	52	0.43	0.04	0.38	0.33
Fruits only	256	0.31	0.02	0.29	0.55
Fruits and grains	66	1.32	0.26	0.33	0.29
Dairy-based	41	0.36	0.15	0.28	0.33
Meat-based meals or snacks	74	0.56	0.03	0.52	0.36
Pasta-based meals or snacks	11	0.79	0.16	0.58	0.96
Vegetable-based mixtures or meals	104	0.67	0.08	0.58	0.49
Cereal bars and breakfast pastries	-	-	-	-	-
Savory snacks	13	3.21	0.63	4.82	4.82
Dry, grain-based desserts	43	1.38	0.12	1.5	1.5
Dry, fruit-based snacks	11	0 *	0 *	0 *	0 *
Juice or drinks	14	0 *	0 *	0 *	0 *
	**Iron (mg)/100 g**				
Cereals, dry and instant	40	41.25	3.29	45.00	7.83
Vegetables only	52	0.39	0.04	0.34	0.30
Fruits only	256	0.29	0.02	0.27	0.50
Fruits and grains	66	1.20	0.24	0.30	0.27
Dairy-based	41	0.32	0.14	0.25	0.30
Meat-based meals or snacks	74	0.51	0.03	0.47	0.33
Pasta-based meals or snacks	11	0.72	0.15	0.53	0.87
Vegetable-based mixtures or meals	104	0.61	0.07	0.53	0.44
Cereal bars and breakfast pastries	-	-	-	-	-
Savory snacks	13	21.43	4.20	32.14	32.14
Dry, grain-based desserts	43	19.74	1.69	21.43	21.43
Dry, fruit-based snacks	11	0 *	0 *	0 *	0 *
Juice/drinks	14	0 *	0 *	0 *	0 *

^a^ Foods marketed for children aged 4–12 months. ^b^ Reference amount customarily consumed (RACC) is a unit measure used by the US Food and Drug Administration to represent the amount of a specified food consumed on average per eating occasion. ^c^ Standard error of the mean (SE). ^d^ Interquartile range (IQR). - Products were not marketed for infants. * The % DV for iron was reported as 0 on the nutrition label.

**Table 3:** Iron content per reference amount customarily consumed (RACC) and per 100 g of toddler ^a^ food and drink products by food category, 2015.

Food Product Category	Iron (mg)/RACC ^b^				
	*N* = 292	Mean	SE ^c^	Median	IQR ^d^
Cereals, dry and instant	-	-	-	-	-
Vegetables only	-	-	-	-	-
Fruits only	49	0.43	0.06	0.44	0.66
Fruits and grains	12	0.80	0.17	0.70	1.00
Dairy-based	59	0.22	0.03	0.18	0.37
Meat-based meals or snacks	22	0.74	0.09	0.68	0.60
Pasta-based meals or snacks	17	1.92	0.31	1.20	2.10
Vegetable-based mixtures or meals	4	2.97	0.71	2.75	1.94
Cereal bars and breakfast pastries	29	1.22	0.15	1.00	1.21
Savory snacks	20	0.78	0.19	0.36	1.10
Dry, grain-based desserts	33	0.50	0.11	0.35	0.88
Dry, fruit-based snacks	36	0.63	0.33	0.21	0.38
Juice/drinks	11	0.10	0.02	0.10	0.20
	**Iron (mg)/100 g**				
Cereals, dry and instant	-	-	-	-	-
Vegetables only	-	-	-	-	-
Fruits only	49	0.35	0.05	0.35	0.53
Fruits and grains	12	0.71	0.15	0.49	0.91
Dairy-based	59	0.20	0.03	0.17	0.33
Meat-based meals or snacks	22	0.43	0.06	0.40	0.35
Pasta-based meals or snacks	17	1.13	0.18	0.71	1.24
Vegetable-based mixtures or meals	4	1.75	0.42	1.62	1.14
Cereal bars and breakfast pastries	29	6.10	0.74	5.00	6.07
Savory snacks	20	5.18	1.28	2.38	7.33
Dry, grain-based desserts	33	6.45	1.08	5.00	12.50
Dry, fruit-based snacks	36	4.19	2.21	1.43	2.54
Juice/drinks	11	0.08	0.02	0.08	0.17

^a^ Foods marketed for children aged 12–36 months. ^b^ Reference amount customarily consumed (RACC) is a unit measure used by the US Food and Drug Administration to represent the amount of a specified food that was consumed on average per eating occasion. ^c^ Standard error of the mean (SE). ^d^ Interquartile range (IQR). - Products were not marketed for toddlers.

## Supplementary Materials

The following are available online at https://www.mdpi.com/2072-6643/12/8/2439/s1: Table S1: Categorization of commercial infant and toddler food and drink products with associated RACC and serving sizes; Table S2: Food categories for commercially available infant and toddler food and drink products.
