# Supplementary material for: Iron Content of Commercially Available Infant and Toddler Foods in the United States, 2015
Source: Nutrients. 2020 Aug 13;12(8):2439. doi: 10.3390/nu12082439 (PMC7469030; doi:10.3390/nu12082439)
Supplement: Supplementary file 1 [file nutrients-12-02439-s001.pdf]

Table S1. Categorization of commercial infant and toddler food and drink products with associated RACC and serving sizes

Food product category	RACC[a] category	RACC, g	Serving size[b], g
Cereals, dry and instant	Cereals, dry and instant	15	15
Vegetables only	Dinners, desserts, fruit, vegetables or soups, ready-to-serve, strained type	110	95
Fruits only	Dinners, desserts, fruit, vegetables or soups, ready-to-serve, strained or junior type. Fruits for toddlers, ready-to-serve	110[c] or 125[d]	113[e] or 120[f]
Fruits and grains	Cereals, prepared, ready-to-serve	110[c] or 170[d]	99[e] or 120[f]
Dairy-based	Dinners, desserts, fruit, vegetables or soups, ready-to-serve, junior type	110	113
Meat-based meals or snacks	Dinners, desserts, fruit, vegetables or soups, ready-to-serve, junior type	110[c] or 170[d]	113[e] or 163[f]
Pasta-based meals or snacks	Dinners, desserts, fruit, vegetables or soups, ready-to-serve, junior type	110[c] or 170[d]	128[e] or 150[f]
Vegetable-based mixtures or meals	Dinners, desserts, fruit, vegetables or soups, ready-to-serve, junior type	110[c] or 170[d]	113[e] or 170[f]
Cereal bars and breakfast pastries	Other cereal and grain products, dry ready-to-eat	20	19
Savory snacks	Dinners, desserts, fruit, vegetables or soups, dry mix	15	8
Dry grain-based desserts	Other cereal and grain products, dry ready-to-eat (e.g., ready-to-eat cereals, cookies, teething biscuits, and toasts)	7[c] or 15[d]	7[e] or 10[f]
Dry fruit-based snacks	Dinners, desserts, fruit, vegetables or soups, dry mix	15	7
Juice/drinks	Juices, all varieties	120	118

[a] Reference Amount Customarily Consumed (RACC) is a unit measure used by the US Food and Drug Administration to represent the amount of the specified food that was consumed on average, per eating occasion.
[b] Median manufacturer serving size as listed on the Nutrition Facts label on products for n=1017 products included in this study by the specific food category.
[c] RACC, g for infants.
[d] RACC, g for toddlers.
[e] Infant products
[f] Toddler products

Table S2: Food categories for commercially available infant and toddler food and drink products[a]

		Bates et al. Food Categories												
**Maalouf et al. Food Categories**[b]	*n*	Cereals, dry and instant	Vegetables only	Fruits only	Fruits and grains	Dairy-based	Meat-based meals or snacks	Pasta-based meals or snacks	Vegetable-based mixtures or meals	Cereal bars and breakfast pastries	Savory snacks	Dry grain-based desserts	Dry fruit-based snacks	Juice/ drinks
Infant														
Vegetables, stages 1–3 (single vegetables, e.g., pureed peas and pureed carrots)	52	0	52	0	0	0	0	0	0	0	0	0	0	0
Dinners, soups, and vegetables, stages 2 and 3 (e.g., vegetables or vegetable, meat, pasta, or soup-based mixed dishes)	189	0	0	0	0	0	74	11	104	0	0	0	0	0
Fruit, stages 1–3 (pureed single or mixed fruit)	256	0	0	256	0	0	0	0	0	0	0	0	0	0
Cereals, dry and instant (e.g., dry rice cereal)	40	40	0	0	0	0	0	0	0	0	0	0	0	0
Mixed grains and fruit, ready-to-serve (e.g., oatmeal and fruit in a jar, ready-to-serve)	78	0	0	0	78	0	0	0	0	0	0	0	0	0
Toddler														
Dinners or meals (vegetable, meat/poultry/fish, and pasta-, pizza- or soup-based mixed dishes)	43	0	0	0	0	0	22	17	4	0	0	0	0	0
Cereal bars and breakfast pastries (e.g., cereal bars, cereal and fruit bars, and cakes or bread)	29	0	0	0	0	0	0	0	0	29	0	0	0	0
Fruit (e.g., pureed fruit mixtures or fruit and vegetable mixtures)	49	0	0	49	0	0	0	0	0	0	0	0	0	0
Dry fruit–based snacks (e.g., freeze-dried yogurt or fruit snacks and dehydrated fruit snacks)	55	0	0	0	0	0	0	0	0	0	0	0	55	0
Sides (e.g. meat sticks, diced vegetables)[a]	5	0	2	0	0	0	3	0	0	0	0	0	0	0
Infant or toddler														
Savory snacks (e.g., crackers and savory rice cakes)	36	0	0	0	0	0	0	0	0	0	36	0	0	0
Dry grain–based desserts (e.g., cookies, sweet biscuits or graham crackers, and sweet rice cakes and puffs)	80	0	0	0	0	0	0	0	0	0	0	80	0	0
Dairy-based desserts (e.g., yogurt with or without fruit)	100	0	0	0	0	100	0	0	0	0	0	0	0	0
Juices and drinks (e.g., single or mixed fruit juices and drinks)	25	0	0	0	0	0	0	0	0	0	0	0	0	25
**Totals**	1037	40	54	305	78	100	99	28	108	29	36	80	55	25

[a]All products included in the database before exclusion are shown (n=1037)
[b]Maalouf J, Cogswell ME, Bates M, Yuan K, Scanlon KS, Pehrsson P, et al. Sodium, sugar, and fat content of complementary infant and toddler foods sold in the United States, 2015. *Am J Clin Nutr*. 2017;105(6):1443-1452.
